# 4-(4-Bromo­phen­yl)-2,6-diphenyl­pyridine

**DOI:** 10.1107/S1600536809049253

**Published:** 2009-11-21

**Authors:** Qun Cao, Yu Xie, Jie Jia, Xiao-Wei Hong

**Affiliations:** a Key Laboratory of Nondestructive Testing (Ministry of Education), Nanchang Hangkong University, Nanchang 330063, People’s Republic of China; b Key Laboratory of Photochemical Conversion and Optoelectronic Materials, TIPC, CAS, Beijing 100190, People’s Republic of China

## Abstract

In the title compound, C_23_H_16_BrN, the three benzene rings show a disrotatory counter-rotating arrangement around the central pyridine ring and are twisted with respect to the pyridine ring with dihedral angles of 19.56 (13), 27.54 (13) and 30.51 (13)°.

## Related literature

For applications of the title compound, see: Verma *et al.* (2007 ▶); Vellis *et al.* (2008 ▶). For related structures, see: Lv & Huang (2008 ▶); Ondrá˘cek *et al.* (1994 ▶). For the synthesis, see: Verma *et al.* (2007 ▶).
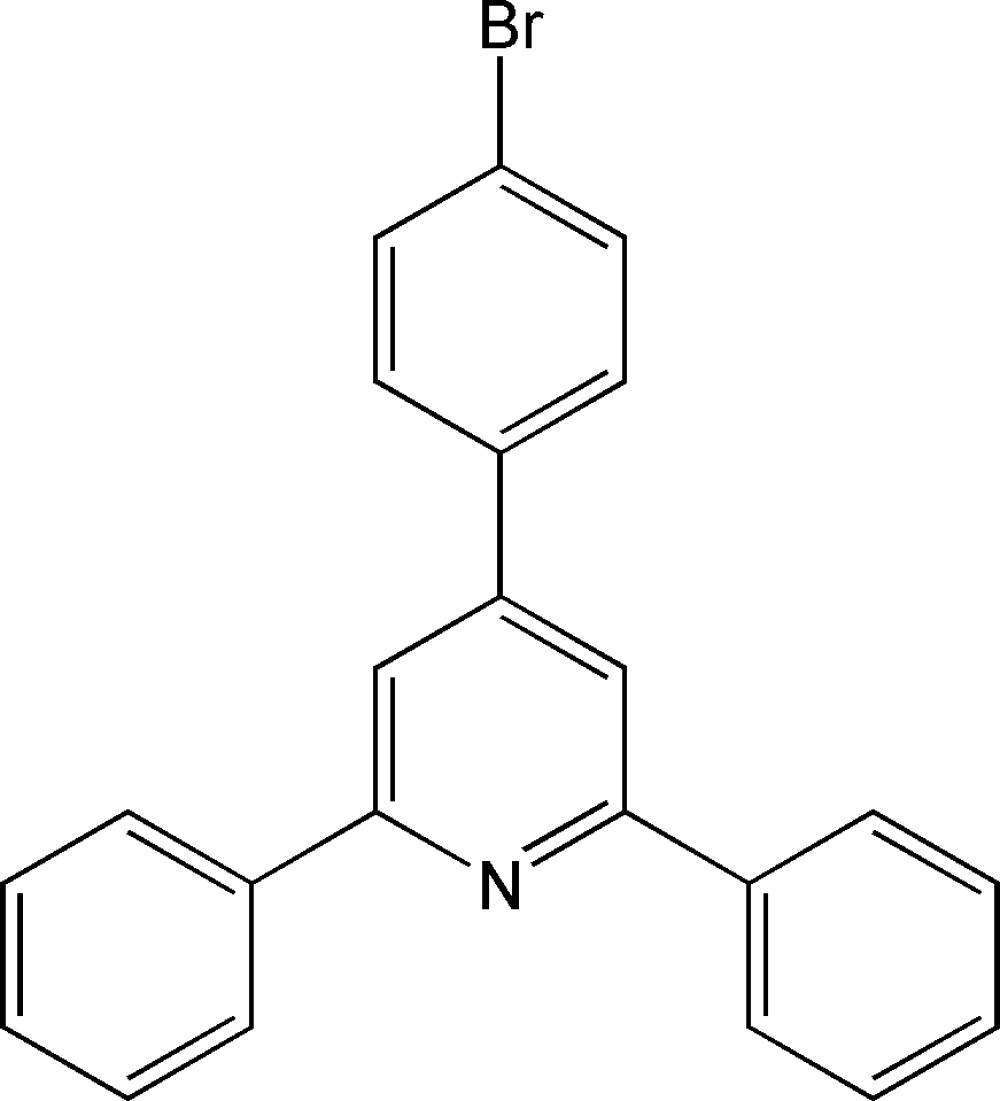


## Experimental

### 

#### Crystal data

C_23_H_16_BrN
*M* *_r_* = 386.28
Monoclinic, 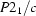

*a* = 8.9837 (4) Å
*b* = 21.5202 (10) Å
*c* = 9.6108 (4) Å
β = 105.5940 (10)°
*V* = 1789.67 (14) Å^3^
*Z* = 4
Mo *K*α radiation
μ = 2.30 mm^−1^
*T* = 293 K
0.30 × 0.22 × 0.20 mm

#### Data collection

Bruker SMART CCD area-detector diffractometer
Absorption correction: multi-scan (*SADABS*; Sheldrick, 1996 ▶) *T*_min_ = 0.542, *T*_max_ = 0.652
13423 measured reflections
4325 independent reflections
2433 reflections with *I* > 2σ(*I*)
*R*_int_ = 0.027

#### Refinement

*R*[*F* ^2^ > 2σ(*F* ^2^)] = 0.038
*wR*(*F* ^2^) = 0.105
*S* = 1.01
4325 reflections
226 parameters
H-atom parameters constrained
Δρ_max_ = 0.42 e Å^−3^
Δρ_min_ = −0.43 e Å^−3^

### 

Data collection: *SMART* (Bruker, 1998 ▶); cell refinement: *SAINT* (Bruker, 1998 ▶); data reduction: *SAINT*; program(s) used to solve structure: *SHELXTL* (Sheldrick, 2008 ▶); program(s) used to refine structure: *SHELXTL*; molecular graphics: *SHELXTL*; software used to prepare material for publication: *SHELXTL*.

## Supplementary Material

Crystal structure: contains datablocks global, I. DOI: 10.1107/S1600536809049253/xu2640sup1.cif

Structure factors: contains datablocks I. DOI: 10.1107/S1600536809049253/xu2640Isup2.hkl

Additional supplementary materials:  crystallographic information; 3D view; checkCIF report

## Figures and Tables

**Fig. 1.**
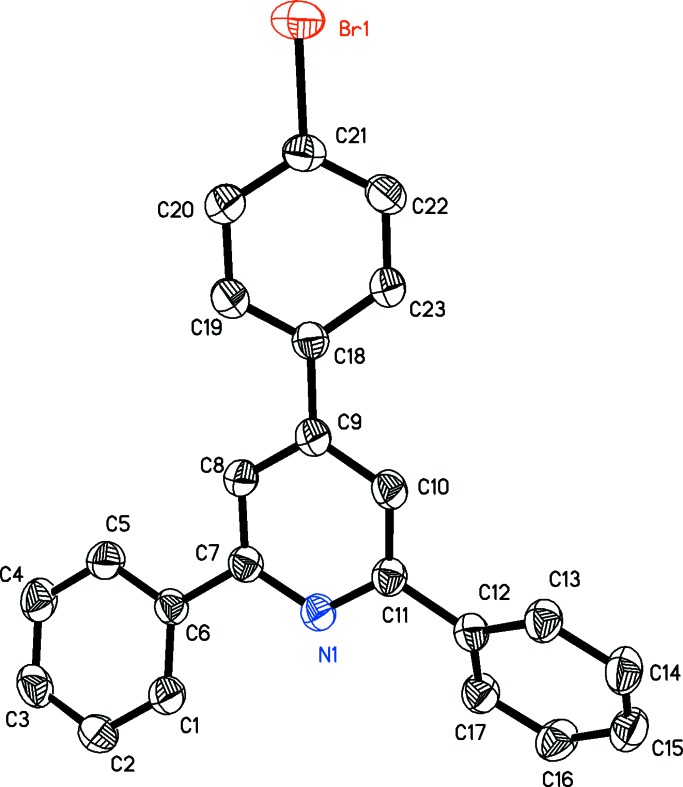
: The molecular structure of compound (I). Displacement ellipsoids are drawn at the 30% probability level. The H atoms are omitted for clarity.

## supplementary crystallographic information

### Comment

The title compound, 4-(4-bromophenyl)-2,6-diphenylpyridine (I), is an useful intermediate in the synthesis of electroluminescent materials or new supramolecules (Verma *et al.*, 2007; Vellis *et al.*, 2008). It has been synthesized previously. We reported its structure here.

In (I) (Fig. 1), the bond lengths and angles are normal and comparable to those observed in reported the compound (Ondrá˘cek *et al.*, 1994; Lv & Huang, 2008). The three phenyl rings display a disrotatory conformation and form different angles with the pyridine ring. The dihedral angles between the pyridine ring and the two phenyls in 2- and 6- position are 19.56 (13) and 27.54 (13) ° respectively, while the phenyl ring in 4- position forms the largest angle with the heterocycle, 30.51 (13)°.

### Experimental

The title compound was prepared by literature method (Verma *et al.*, 2007). Colorless single crystals suitable for X-ray diffraction were obtained from the solution of dichloromethane by vapor diffusion with hexane.

### Refinement

All H atoms were positioned geomertrically and treated as riding (C—H = 0.93 Å) with *U*_iso_(H) = 1.2*U*_eq_(C).

### Crystal data

**Table:**

C_23_H_16_BrN	*F*(000) = 784
*M**_r_* = 386.28	*D*_x_ = 1.434 Mg m^−^^3^
Monoclinic, *P*2_1_/*c*	Mo *K*α radiation, λ = 0.71073 Å
Hall symbol: -P 2ybc	Cell parameters from 987 reflections
*a* = 8.9837 (4) Å	θ = 2.9–25.1°
*b* = 21.5202 (10) Å	µ = 2.30 mm^−^^1^
*c* = 9.6108 (4) Å	*T* = 293 K
β = 105.594 (1)°	Block, colorless
*V* = 1789.67 (14) Å^3^	0.30 × 0.22 × 0.20 mm
*Z* = 4	

### Data collection

**Table:**

Bruker SMART CCD area-detector diffractometer	4325 independent reflections
Radiation source: fine-focus sealed tube	2433 reflections with *I* > 2σ(*I*)
graphite	*R*_int_ = 0.027
φ and ω scans	θ_max_ = 28.3°, θ_min_ = 1.9°
Absorption correction: multi-scan (*SADABS*; Sheldrick, 1996)	*h* = −11→11
*T*_min_ = 0.542, *T*_max_ = 0.652	*k* = −28→27
13423 measured reflections	*l* = −11→12

### Refinement

**Table:**

Refinement on *F*^2^	Primary atom site location: structure-invariant direct methods
Least-squares matrix: full	Secondary atom site location: difference Fourier map
*R*[*F*^2^ > 2σ(*F*^2^)] = 0.038	Hydrogen site location: inferred from neighbouring sites
*wR*(*F*^2^) = 0.105	H-atom parameters constrained
*S* = 1.01	*w* = 1/[σ^2^(*F*_o_^2^) + (0.0449*P*)^2^ + 0.3089*P*] where *P* = (*F*_o_^2^ + 2*F*_c_^2^)/3
4325 reflections	(Δ/σ)_max_ = 0.001
226 parameters	Δρ_max_ = 0.42 e Å^−^^3^
0 restraints	Δρ_min_ = −0.43 e Å^−^^3^

### Special details

**Table:**

Geometry. All e.s.d.'s (except the e.s.d. in the dihedral angle between two l.s. planes) are estimated using the full covariance matrix. The cell e.s.d.'s are taken into account individually in the estimation of e.s.d.'s in distances, angles and torsion angles; correlations between e.s.d.'s in cell parameters are only used when they are defined by crystal symmetry. An approximate (isotropic) treatment of cell e.s.d.'s is used for estimating e.s.d.'s involving l.s. planes.
Refinement. Refinement of *F*^2^ against ALL reflections. The weighted *R*-factor *wR* and goodness of fit *S* are based on *F*^2^, conventional *R*-factors *R* are based on *F*, with *F* set to zero for negative *F*^2^. The threshold expression of *F*^2^ > σ(*F*^2^) is used only for calculating *R*-factors(gt) *etc*. and is not relevant to the choice of reflections for refinement. *R*-factors based on *F*^2^ are statistically about twice as large as those based on *F*, and *R*- factors based on ALL data will be even larger.

### Fractional atomic coordinates and isotropic or equivalent isotropic displacement parameters (Å^2^)

**Table:**

	*x*	*y*	*z*	*U*_iso_*/*U*_eq_	
Br1	0.28459 (4)	0.440827 (13)	0.83895 (4)	0.08918 (16)	
N1	−0.0501 (2)	0.82145 (8)	0.9831 (2)	0.0538 (5)	
C7	−0.1196 (2)	0.76960 (11)	1.0130 (2)	0.0518 (6)	
C12	0.1459 (2)	0.87312 (11)	0.8977 (3)	0.0530 (6)	
C21	0.2129 (3)	0.52227 (11)	0.8610 (3)	0.0597 (6)	
C23	0.2619 (3)	0.62976 (12)	0.9073 (3)	0.0599 (6)	
H23A	0.3310	0.6628	0.9268	0.072*	
C16	0.2240 (3)	0.97801 (12)	0.9653 (3)	0.0733 (7)	
H16A	0.2220	1.0125	1.0230	0.088*	
C9	0.0510 (3)	0.70294 (10)	0.9250 (3)	0.0527 (6)	
C11	0.0680 (3)	0.81496 (10)	0.9238 (3)	0.0527 (6)	
C10	0.1200 (3)	0.75706 (11)	0.8935 (3)	0.0570 (6)	
H10A	0.2019	0.7545	0.8517	0.068*	
C8	−0.0713 (3)	0.71067 (10)	0.9864 (3)	0.0556 (6)	
H8A	−0.1212	0.6759	1.0098	0.067*	
C19	0.0068 (3)	0.59007 (11)	0.8679 (3)	0.0617 (6)	
H19A	−0.0978	0.5962	0.8591	0.074*	
C18	0.1063 (3)	0.64034 (10)	0.8985 (3)	0.0526 (6)	
C17	0.1437 (3)	0.92504 (11)	0.9818 (3)	0.0629 (7)	
H17A	0.0876	0.9243	1.0502	0.076*	
C15	0.3066 (3)	0.98002 (13)	0.8645 (3)	0.0718 (8)	
H15A	0.3629	1.0154	0.8555	0.086*	
C13	0.2264 (3)	0.87648 (12)	0.7932 (3)	0.0620 (6)	
H13A	0.2266	0.8425	0.7335	0.074*	
C5	−0.3104 (3)	0.73186 (13)	1.1431 (3)	0.0683 (7)	
H5A	−0.2590	0.6939	1.1585	0.082*	
C20	0.0587 (3)	0.53108 (11)	0.8502 (3)	0.0628 (6)	
H20A	−0.0096	0.4977	0.8313	0.075*	
C6	−0.2546 (3)	0.77920 (11)	1.0728 (3)	0.0525 (6)	
C1	−0.3309 (3)	0.83534 (12)	1.0563 (3)	0.0681 (7)	
H1B	−0.2942	0.8681	1.0117	0.082*	
C2	−0.4611 (3)	0.84380 (13)	1.1047 (3)	0.0772 (8)	
H2A	−0.5110	0.8821	1.0926	0.093*	
C14	0.3058 (3)	0.92962 (13)	0.7769 (3)	0.0716 (8)	
H14A	0.3591	0.9313	0.7063	0.086*	
C4	−0.4407 (3)	0.74032 (14)	1.1904 (3)	0.0748 (8)	
H4A	−0.4774	0.7080	1.2361	0.090*	
C22	0.3151 (3)	0.57114 (12)	0.8876 (3)	0.0640 (7)	
H22A	0.4187	0.5648	0.8924	0.077*	
C3	−0.5166 (3)	0.79637 (13)	1.1701 (3)	0.0731 (8)	
H3A	−0.6054	0.8019	1.2009	0.088*	

### Atomic displacement parameters (Å^2^)

**Table:**

	*U*^11^	*U*^22^	*U*^33^	*U*^12^	*U*^13^	*U*^23^
Br1	0.0970 (3)	0.0623 (2)	0.1124 (3)	0.02012 (15)	0.0353 (2)	0.00041 (16)
N1	0.0535 (11)	0.0557 (11)	0.0563 (13)	−0.0001 (9)	0.0215 (9)	−0.0006 (9)
C7	0.0517 (13)	0.0565 (13)	0.0503 (15)	0.0016 (10)	0.0190 (11)	0.0046 (11)
C12	0.0506 (13)	0.0571 (14)	0.0539 (15)	−0.0012 (10)	0.0185 (11)	−0.0006 (11)
C21	0.0681 (16)	0.0547 (14)	0.0591 (16)	0.0088 (12)	0.0220 (13)	0.0018 (11)
C23	0.0553 (14)	0.0605 (15)	0.0697 (17)	−0.0020 (11)	0.0268 (13)	0.0006 (12)
C16	0.0888 (19)	0.0562 (15)	0.078 (2)	−0.0075 (14)	0.0275 (16)	−0.0077 (13)
C9	0.0499 (13)	0.0569 (13)	0.0530 (15)	−0.0002 (10)	0.0169 (11)	0.0002 (11)
C11	0.0538 (13)	0.0556 (13)	0.0518 (15)	−0.0034 (10)	0.0193 (11)	−0.0010 (11)
C10	0.0540 (14)	0.0617 (14)	0.0616 (16)	−0.0008 (11)	0.0264 (12)	−0.0023 (12)
C8	0.0550 (14)	0.0519 (13)	0.0642 (17)	−0.0024 (11)	0.0233 (12)	0.0033 (11)
C19	0.0509 (13)	0.0642 (15)	0.0719 (18)	0.0019 (12)	0.0199 (12)	−0.0034 (13)
C18	0.0546 (14)	0.0536 (13)	0.0534 (15)	0.0016 (11)	0.0211 (11)	0.0022 (11)
C17	0.0676 (15)	0.0623 (15)	0.0651 (18)	−0.0021 (12)	0.0283 (13)	−0.0044 (12)
C15	0.0720 (17)	0.0625 (16)	0.082 (2)	−0.0154 (13)	0.0233 (16)	0.0028 (14)
C13	0.0646 (15)	0.0619 (15)	0.0654 (17)	−0.0070 (12)	0.0276 (13)	−0.0063 (12)
C5	0.0726 (17)	0.0617 (15)	0.080 (2)	0.0029 (13)	0.0366 (15)	0.0087 (13)
C20	0.0646 (16)	0.0544 (14)	0.0713 (18)	−0.0029 (12)	0.0214 (13)	−0.0035 (12)
C6	0.0484 (13)	0.0567 (13)	0.0560 (15)	0.0003 (10)	0.0199 (11)	−0.0019 (11)
C1	0.0663 (16)	0.0598 (15)	0.088 (2)	0.0006 (12)	0.0375 (15)	0.0012 (14)
C2	0.0694 (17)	0.0671 (17)	0.106 (2)	0.0104 (13)	0.0426 (17)	−0.0017 (16)
C14	0.0718 (17)	0.0760 (18)	0.076 (2)	−0.0115 (13)	0.0353 (15)	0.0028 (15)
C4	0.0745 (18)	0.0799 (19)	0.083 (2)	−0.0101 (15)	0.0439 (16)	0.0053 (15)
C22	0.0567 (14)	0.0686 (17)	0.0702 (18)	0.0105 (12)	0.0231 (13)	0.0039 (13)
C3	0.0576 (16)	0.087 (2)	0.084 (2)	0.0001 (14)	0.0350 (15)	−0.0095 (16)

### Geometric parameters (Å, °)

**Table:**

Br1—C21	1.899 (2)	C19—C20	1.379 (3)
N1—C11	1.340 (3)	C19—C18	1.383 (3)
N1—C7	1.347 (3)	C19—H19A	0.9300
C7—C8	1.386 (3)	C17—H17A	0.9300
C7—C6	1.490 (3)	C15—C14	1.372 (4)
C12—C17	1.382 (3)	C15—H15A	0.9300
C12—C13	1.389 (3)	C13—C14	1.379 (3)
C12—C11	1.488 (3)	C13—H13A	0.9300
C21—C20	1.374 (3)	C5—C4	1.377 (3)
C21—C22	1.374 (4)	C5—C6	1.388 (3)
C23—C22	1.380 (3)	C5—H5A	0.9300
C23—C18	1.397 (3)	C20—H20A	0.9300
C23—H23A	0.9300	C6—C1	1.377 (3)
C16—C15	1.371 (4)	C1—C2	1.383 (3)
C16—C17	1.381 (3)	C1—H1B	0.9300
C16—H16A	0.9300	C2—C3	1.362 (4)
C9—C8	1.390 (3)	C2—H2A	0.9300
C9—C10	1.390 (3)	C14—H14A	0.9300
C9—C18	1.481 (3)	C4—C3	1.374 (4)
C11—C10	1.389 (3)	C4—H4A	0.9300
C10—H10A	0.9300	C22—H22A	0.9300
C8—H8A	0.9300	C3—H3A	0.9300
			
C11—N1—C7	118.03 (19)	C16—C17—H17A	119.6
N1—C7—C8	122.17 (19)	C12—C17—H17A	119.6
N1—C7—C6	116.1 (2)	C16—C15—C14	119.6 (2)
C8—C7—C6	121.7 (2)	C16—C15—H15A	120.2
C17—C12—C13	118.2 (2)	C14—C15—H15A	120.2
C17—C12—C11	120.1 (2)	C14—C13—C12	120.8 (2)
C13—C12—C11	121.7 (2)	C14—C13—H13A	119.6
C20—C21—C22	121.2 (2)	C12—C13—H13A	119.6
C20—C21—Br1	118.97 (19)	C4—C5—C6	120.9 (3)
C22—C21—Br1	119.85 (19)	C4—C5—H5A	119.5
C22—C23—C18	121.2 (2)	C6—C5—H5A	119.5
C22—C23—H23A	119.4	C21—C20—C19	119.0 (2)
C18—C23—H23A	119.4	C21—C20—H20A	120.5
C15—C16—C17	120.4 (2)	C19—C20—H20A	120.5
C15—C16—H16A	119.8	C1—C6—C5	117.8 (2)
C17—C16—H16A	119.8	C1—C6—C7	120.6 (2)
C8—C9—C10	116.2 (2)	C5—C6—C7	121.6 (2)
C8—C9—C18	121.4 (2)	C6—C1—C2	121.1 (2)
C10—C9—C18	122.3 (2)	C6—C1—H1B	119.4
N1—C11—C10	122.1 (2)	C2—C1—H1B	119.4
N1—C11—C12	116.48 (19)	C3—C2—C1	120.3 (3)
C10—C11—C12	121.3 (2)	C3—C2—H2A	119.8
C11—C10—C9	120.8 (2)	C1—C2—H2A	119.8
C11—C10—H10A	119.6	C15—C14—C13	120.2 (3)
C9—C10—H10A	119.6	C15—C14—H14A	119.9
C7—C8—C9	120.7 (2)	C13—C14—H14A	119.9
C7—C8—H8A	119.7	C3—C4—C5	120.2 (3)
C9—C8—H8A	119.7	C3—C4—H4A	119.9
C20—C19—C18	121.7 (2)	C5—C4—H4A	119.9
C20—C19—H19A	119.1	C21—C22—C23	119.1 (2)
C18—C19—H19A	119.1	C21—C22—H22A	120.4
C19—C18—C23	117.7 (2)	C23—C22—H22A	120.4
C19—C18—C9	121.4 (2)	C2—C3—C4	119.6 (2)
C23—C18—C9	120.9 (2)	C2—C3—H3A	120.2
C16—C17—C12	120.7 (2)	C4—C3—H3A	120.2
			
C11—N1—C7—C8	1.0 (3)	C13—C12—C17—C16	−2.2 (4)
C11—N1—C7—C6	−177.4 (2)	C11—C12—C17—C16	175.7 (2)
C7—N1—C11—C10	−0.3 (4)	C17—C16—C15—C14	1.8 (4)
C7—N1—C11—C12	−177.6 (2)	C17—C12—C13—C14	2.0 (4)
C17—C12—C11—N1	26.4 (3)	C11—C12—C13—C14	−175.9 (2)
C13—C12—C11—N1	−155.7 (2)	C22—C21—C20—C19	−0.6 (4)
C17—C12—C11—C10	−150.9 (2)	Br1—C21—C20—C19	179.22 (19)
C13—C12—C11—C10	27.0 (4)	C18—C19—C20—C21	−1.1 (4)
N1—C11—C10—C9	−0.4 (4)	C4—C5—C6—C1	2.0 (4)
C12—C11—C10—C9	176.8 (2)	C4—C5—C6—C7	−176.2 (3)
C8—C9—C10—C11	0.4 (3)	N1—C7—C6—C1	19.2 (3)
C18—C9—C10—C11	−178.1 (2)	C8—C7—C6—C1	−159.2 (2)
N1—C7—C8—C9	−1.0 (4)	N1—C7—C6—C5	−162.6 (2)
C6—C7—C8—C9	177.3 (2)	C8—C7—C6—C5	19.0 (4)
C10—C9—C8—C7	0.2 (4)	C5—C6—C1—C2	−1.6 (4)
C18—C9—C8—C7	178.7 (2)	C7—C6—C1—C2	176.6 (3)
C20—C19—C18—C23	1.6 (4)	C6—C1—C2—C3	−0.1 (5)
C20—C19—C18—C9	−176.7 (2)	C16—C15—C14—C13	−2.0 (4)
C22—C23—C18—C19	−0.6 (4)	C12—C13—C14—C15	0.1 (4)
C22—C23—C18—C9	177.8 (2)	C6—C5—C4—C3	−0.8 (4)
C8—C9—C18—C19	30.6 (4)	C20—C21—C22—C23	1.6 (4)
C10—C9—C18—C19	−151.0 (2)	Br1—C21—C22—C23	−178.2 (2)
C8—C9—C18—C23	−147.7 (2)	C18—C23—C22—C21	−0.9 (4)
C10—C9—C18—C23	30.7 (3)	C1—C2—C3—C4	1.3 (5)
C15—C16—C17—C12	0.4 (4)	C5—C4—C3—C2	−0.9 (5)
